# Enhanced MICP for Soil Improvement and Heavy Metal Remediation: Insights from Landfill Leachate-Derived Ureolytic Bacterial Consortium

**DOI:** 10.3390/microorganisms13010174

**Published:** 2025-01-15

**Authors:** Armstrong Ighodalo Omoregie, Fock-Kui Kan, Hazlami Fikri Basri, Muhammad Oliver Ensor Silini, Adharsh Rajasekar

**Affiliations:** 1 Research Centre for Borneo Regionalism and Conservation, School of Built Environment, University of Technology Sarawak, No. 1 Jalan University, Sibu 96000, Malaysia; armstrong@uts.edu.my (A.I.O.); fockkuikan@uts.edu.my (F.-K.K.); 2 Department of Water and Environmental Engineering, Faculty of Civil Engineering, Universiti Teknologi Malaysia, Johor Bahru 81310, Malaysia; hazlami@utm.my; 3 Key Laboratory of Meteorological Disaster, Ministry of Education (KLME)/Joint International Research Laboratory of Climate and Environmental Change (ILCEC)/Collaborative Innovation Centre on Forecast and Evaluation of Meteorological Disasters (CIC-FEMD), Nanjing University of Information Science & Technology, Nanjing 210044, China

**Keywords:** microbial-induced calcium carbonate precipitation, soil stabilization, heavy metal removal, ureolytic bacteria, landfill leachate

## Abstract

This study investigates the potential of microbial-induced calcium carbonate precipitation (MICP) for soil stabilization and heavy metal immobilization, utilizing landfill leachate-derived ureolytic consortium. Experimental conditions identified yeast extract-based media as most effective for bacterial growth, urease activity, and calcite formation compared to nutrient broth and brown sugar media. Optimal MICP conditions, at pH 8–9 and 30 °C, supported the most efficient biomineralization. The process facilitated the removal of Cd^2+^ (99.10%) and Ni^2+^ (78.33%) while producing stable calcite crystals that enhanced soil strength. Thermal analyses (thermogravimetric analysis (TGA) and differential scanning calorimetry (DSC)) confirmed the successful production of CaCO_3_ and its role in improving soil stability. DSC analysis revealed endothermic and exothermic peaks, including a significant exothermic peak at 444 °C, corresponding to the thermal decomposition of CaCO_3_ into CO_2_ and CaO, confirming calcite formation. TGA results showed steady weight loss, consistent with the breakdown of CaCO_3_, supporting the formation of stable carbonates. The MICP treatment significantly increased soil strength, with the highest surface strength observed at 440 psi, correlating with the highest CaCO_3_ content (18.83%). These findings underscore the effectiveness of MICP in soil stabilization, pollutant removal, and improving geotechnical properties.

## 1. Introduction

One standard method of managing waste is the practice of landfilling. However, it has significant environmental risks, including the emission of greenhouse gases and the production of leachate [1]. In landfill risk assessments, the re-release of leachate is regarded as the most critical danger, particularly when sites are mismanaged [2,3]. A hazardous liquid known as landfill leachate is created when too much precipitation seeps through the waste materials in a landfill, possibly contaminating the surface and groundwater [4,5]. It includes a variety of organic and inorganic contaminants, including phenolic compounds, heavy metals, polycyclic aromatic hydrocarbons (PAHs), agrochemicals, and pharmaceutical residues [6,7]. These toxins pose severe threats to the surrounding ecosystems and environmental integrity due to the lack of appropriate management techniques. Traditional methods of treating landfill leachate include physical filtration and chemical processes like coagulation and oxidation. However, these methods do not always entirely remove dissolved contaminants like organic materials and heavy metals [8,9]. Nevertheless, these techniques frequently result in secondary waste that is expensive and energy-intensive and do not completely address the complex pollutants found in landfill leachate, underscoring the need for creative and long-lasting solutions.

Using ureolytic bacteria for calcite biomineralization is a promising environmental engineering approach. These microbes (*Sporosarcina* sp., *Bacillus* sp.) use the urease enzyme (EC 3.5.1.5) to break down urea (CO(NH_2_)_2_) into ammonia (NH_3_) and carbonic acid (H_2_CO_3_) [10,11]. Carbonic acid then dissociates into carbonate ions (CO_3_^2−^), which precipitate as calcium carbonate (CaCO_3_), or calcite, in the presence of calcium ions (Ca^2+^) [12,13]. This stable, crystalline calcite can fortify soils and immobilize pollutants, making microbial-induced carbonate precipitation (MICP) valuable for soil stability, bioremediation, and mortar repair [14,15,16,17]. MICP bacteria can infiltrate the fine interstices of materials, facilitating CaCO_3_ precipitation and addressing challenges in soil stabilization, environmental remediation, and sustainable construction. The urease enzyme also plays a crucial role in nitrogen metabolism, aiding in the recycling of nitrogen for various biological processes [18].

Biostimulation, which increases the functioning of native microbial communities by supplying specific nutrients or substrates, is a noteworthy development in MICP technology [19,20]. Using this method, ureolytic bacteria may work much more efficiently throughout the biomineralization process [21,22]. By their very nature, employing native bacteria is more sustainable and efficient than introducing alien bacterial strains (bioaugmentation), which can be challenging to adapt to the local environment [20]. Since native bacteria are already adapted to their surroundings, using them in biomineralization processes is a more practical way to produce long-term, ecologically beneficial results [23,24]. Over the years, much research has been conducted on biostimulation for MICP [19,20,22,25,26]. However, the research on enriching ureolytic bacteria in landfill leachate for calcite biomineralization is severely lacking. This process may be optimized by stimulating native bacteria to increase their ureolytic activity. Biostimulation can significantly increase bacterial activity by providing specific nutrients or substrates, improving calcite precipitation, and immobilizing pollutants more effectively [27]. Comparing this approach to others that use non-native bacterial strains, it is more economical, reduces ecological hazards, and improves sustainability. Biostimulation is an efficient and ecologically friendly method of treating landfill leachate and other polluted sites because local bacteria are naturally suited to the unique circumstances of the landfill environment.

This study investigates the potential of MICP, employing native ureolytic bacteria from landfill leachate, as a sustainable alternative. The objectives were to (i) optimize growth conditions for ureolytic bacteria derived from landfill leachate; (ii) evaluate the efficiency of MICP for heavy metal removal and soil stabilization; (iii) analyze the structural and thermal properties of biocemented soil; and (iv) compare the proposed method with traditional soil remediation techniques. The mineral composition, microstructure, and thermal stability of the biocemented samples were analyzed using X-ray diffraction (XRD), Fourier-transform infrared spectroscopy (FTIR), thermogravimetric analysis (TGA), scanning electron microscopy (SEM) and energy-dispersive X-ray spectroscopy (EDS). Mechanical testing and effluent analysis assessed heavy metal immobilization efficiency and soil stability enhancement. These investigations aim to elucidate the biochemical mechanisms driving MICP biocementation in landfill leachate-derived bacteria to contribute to sustainable environmental remediation and waste-to-resource initiatives.

## 2. Materials and Methods

### 2.1. Landfill Leachate Sampling Collection Site

Leachate samples were collected from the Seelong sanitary landfill in Kulai, Johor, Malaysia (1°39′35.5″ N, 103°43′11.2″ E). This site, operated by SWM Environment Sdn Bhd, Johor Bahru, Malaysia features Malaysia’s first landfill gas generation facility in a tropical rainforest climate with temperatures of 27–32 °C and humidity levels around 80%. Approximately 10 L of leachate was collected from areas near the landfill’s base, at 2–4 m depths, using sterile containers to maintain sample integrity. The collected leachate was dark brown and had a strong smell, which is a characteristic of leachate produced during the decomposition of organic waste in landfills. Samples were immediately stored on ice, transported to the laboratory, and refrigerated at 4 °C to preserve chemical and biological properties until further processing. Upon arriving at the lab, duplicate samples weighing 1 L were moved to the IPASA Center for Environmental and Water Security at the Research Institute for Sustainable Environment in Johor Bahru, Malaysia. The necessary physicochemical analyses were carried out there using standard procedures. Table S1 displays the classification’s results.

### 2.2. Chemicals and Reagents Utilized

This study utilized several analytical-grade chemicals. Urea (CO(NH_2_)_2_), calcium chloride (CaCl_2_), ammonium chloride (NH_4_Cl), and nickel chloride (NiCl_2_) were sourced from Take It Global Sdn. Bhd., Pulau Pinang, Malaysia. Sodium hydroxide (NaOH), sodium chloride (NaCl), and hydrochloric acid (HCl) were obtained from Bendosen Laboratory Chemicals, Darul Ehsan, Malaysia. Central Sugars Refinery Sdn provided brown sugar, Bhd., Selangor, Malaysia, and nutrient broth was supplied by Himedia Laboratories Pvt. Ltd., Maharashtra, India. Angel Yeast Co., Ltd., Yichang, China, supplied yeast extract.

### 2.3. Enrichment Culturing of Leachate Sample

Three media, namely, yeast extract medium (YEM), nutrient broth medium (NBM), and brown sugar medium (BSM) were used to enhance native ureolytic bacterial cells from leachate wastewater. The media compositions (Table 1) were adapted from Omoregie et al. [28]. Each medium was prepared in separate shake flasks and sterilized (Hirayama-HVE-50, Kasukabe-Shi Saitama, Japan) at 121 °C for 50 min. The pH values were adjusted using 0.1 M HCl or NaOH. The concentration of NiCl_2_ (0.02 g/L) added for enrichment culturing is relatively low but needed to promote urease activity without significantly affecting bacterial proliferation.

First, 12.5 mL of leachate samples were added to 112.5 mL of each medium to start the enrichment process. The pH of the enriched culture was adjusted to 7.5, and the process was conducted under aseptic conditions at 32 °C and 150 rpm for 72 h The post-enrichment procedure was repeated with fresh media to target ureolytic microorganisms. The growth (OD_600_), pH, urease activity (Christensen medium, Fisher Scientific (M) Sdn Bhd, Shah Alam, Malaysia), biomineralization test, and electric conductivity were measured (see Figure S4) following Omoregie et al. [29]. Urease production was checked using heavy inoculation on urea agar base medium, which was incubated at 32 °C for 48 h.

(1) $$Urease\;activity\;\left(mM\;urea\;hydrolysed\right)=\frac{\left(EC6-EC1\right)}{6}\times 10\times 11.11$$

whereas *EC*_6_ shows the conductivity measurement at 7 min, and *EC*_1_ represents the conductivity measurement acquired in less than 1 min [28].

The bacterial community from the enriched culture was analyzed using 16S rRNA sequencing by Apical Scientific Sdn. Bhd. (Seri Kembangan, Malaysia). The DADA2 pipeline was used for high-resolution taxonomic assignment, and sequences were processed using the SILVA nr database v138.1. Nevertheless, subsequent experiments were carried out exclusively with the enriched bacterial ureolytic consortium obtained from the successful enrichment process.

### 2.4. Temperature and pH Impact on Enriched Ureolytic Culture

The growth performance of enriched ureolytic bacterial culture was assessed at five temperatures (10 to 50 °C, at 10 °C intervals) and pH values ranging from 6 to 11 using yeast extract medium (YEM). The starting pH for temperature impact experiments was set at 7.0. Sterile 125 mL shake flasks were filled with YEM and inoculated with 10% (*v*/*v*) freshly prepared bacterial starter, incubating for 24 h at 150 rpm in a CERTOMAT^®^ CT plus (Sartorius, Göttingen, Germany). Changes in pH during incubation were monitored due to expected alterations from urease activity.

In a separate experiment, the effect of initial pH on bacterial growth was examined at 7.0, adjusting with 1 M HCl or NaOH. Incubation was carried out for 24 h at the optimal temperature. Biomass, pH, and urease activity were measured post-incubation for both experiments. Bacterial biomass was quantified by measuring optical density at 600 nm (OD_600_) using a UV–Visible spectrophotometer (GENESYS^TM^ 20, Thermo Fisher Scientific, Waltham, MA, USA) with blank samples (uninoculated growth medium) used for calibration. Six replicates were used for each condition to ensure reproducibility.

### 2.5. Preliminary Test on Heavy Metals Removal

A preliminary test was conducted to evaluate the removal of heavy metals (CuCl_2_, CdCl_2_, NiCl_2_, and CrCl_2_) by ureolytic bacteria from landfill leachate. A mixture of 15 mL saline, 5 mL bacterial suspension, urea (2 g/L), CaCl_2_ (4 g/L), and individual heavy metals (10 mg/L) was prepared with a final volume of 20 mL. Cultures were incubated at 30 °C for 24 h. Metal concentrations were measured using flame atomic absorption spectrophotometry (FAAS) with an iCE 3000 series spectrometer (Thermo Fisher Scientific, Waltham, MA, USA). Calibration curves for each metal were created using certified standard solutions, and detection limits were set based on the instrument’s specifications. Control experiments without bacterial inoculum were conducted to account for abiotic metal removal. The metal removal efficiency was calculated using the following equation:

(2) $$Removal\;efficiency\;\left(\%\right)=\frac{\left(Ri-Rf\right)}{Ri}\times 100$$

where R*i* and R*f* represent the initial and final concentrations of heavy metals (mg/L), respectively [30]. Chemicals such as copper chloride (CuCl_2_), cadmium chloride (CdCl_2_), nickel chloride (NiCl_2_), and chromium chloride (CrCl_2_) were acquired from HMBG Chemicals, Wisma Rampai, Malaysia. All heavy metals used in the study had a purity of >99%.

### 2.6. Soil Biocementation Treatment

Industrial-grade pure silica sand, supplied by a local vendor, was used for the MICP treatment. The sand’s particle size distribution was D_10_ = 0.60 mm, D_30_ = 1.19 mm, D_60_ = 1.57 mm, and Dmax = 2.23 mm (Figure S5). The sand’s curvature coefficient (Cc) was 0.93, and its uniformity coefficient (Cu) was 1.87. According to the Unified Soil Classification System (USCS), the sand is classified as poorly graded sand (SP) [31]. The selection of industrial-grade silica sand for MICP treatment was based on its high uniformity coefficient (Cu) and well-graded particle size distribution, promoting efficient percolation and minimizing clogging risk during treatment [29]. These characteristics make the sand ideal for consistent biocementation. Other properties included a pH of 6.56, moisture content of 2.92%, specific gravity (Gs) of 2.65 kg/m^3^, maximum dry density (ρd max) of 1.70 mg/m^3^, and minimum dry density (ρd min) of 1.50 mg/m^3^. The sand was compacted in the column using a standard proctor compaction method, ensuring uniform density for reproducible results. Plastic cylindrical containers were prepared as soil columns for the MICP treatment, each featuring five drainage holes (12 cm height, 7 cm diameter) at the base. Scholars have previously employed plastic cylindrical containers as soil columns for MICP treatment tests [32,33]. A non-woven fabric layer was placed at the bottom of each column to prevent sand loss, and 650 g of compacted dry silica sand was added.

The MICP treatment was performed by percolating bacterial consortia and cementation solutions through the sand columns at a 10 mL/min-controlled flow rate. The flow rate was monitored for each column to ensure consistent CaCO_3_ precipitation and minimize variability between experiments. On the first, third, fifth, and eighth days of MICP treatment, 100 mL of ureolytic bacterial culture (biomass concentration of 1.2 × 10^8^ CFU/mL, and 15.07 mM urea hydrolyzed/min) was introduced to each column. Bacterial culture treatment was administered daily, while cementation fixation with 80 mL of 0.75 M CaCl_2_ and 10 g/L of NH_4_Cl was applied only on the first day. From day one to day eight, a daily treatment solution of 100 mL was added, consisting of 2 g/L yeast extract, 0.75 M urea, 0.75 M CaCl_2_, and 10 g/L NH_4_Cl. Upon completion of the biocementation cycles, a final 100 mL flush of 0.05 M sodium chloride solution was applied to remove residual chemicals.

### 2.7. Measurement of Effluent, Surface Strength Test, and CaCO_3_ Content

Effluent samples were collected every 24 h during the MICP treatment and transferred to sterile 50 mL plastic centrifuge tubes (Falcon^®^ Conical, New York, NY, USA) for pH measurement. The pH of each sample was determined using a SevenEasy^TM^ pH meter (Mettler Toledo, Columbus, OH, USA) by inserting the probe directly into the effluent sample. Ammonium concentrations in the effluent were analyzed using salicylate, which converts ammonia ions into an intense blue indophenol-like dye [34]. The absorbance of the dye was measured using UV–Vis spectroscopy, following standard procedures. Before analysis, the effluent samples were filtered through 0.45 μm Millipore filters and concentrated.

After the curing period, surface strength was determined using a pocket penetrometer. The surface strength test was limited to the top layer of 6.35 mm (0.25 inches), but multiple measurements were conducted across different points on the surface to ensure representative results. Due to the shallow penetration, this measurement only assesses surface strength and may not reflect the overall internal strength of the biocemented column [29]. For CaCO_3_ content measurement, 100 g of dried soil was treated with diluted 2 M HCl to dissolve carbonate crystals. The acid wash method was used, rinsing the sample with deionized water and filtering through the Whatman filter paper [35]. The soil was oven-dried at 60 °C for 3 h.

### 2.8. SEM-EDS, XRD, and FTIR Analyses

Scanning electron microscopy (SEM) and energy-dispersive X-ray spectroscopy (EDS) were used to identify and quantify biocrystals in MICP-treated soil samples. To prevent charge accumulation and improve image quality, the samples were dried at 60 °C, sputter-coated with carbon or gold, and analyzed using a Hitachi TM4000 SEM (Hitachi High-Tech Corporation, Hitachi City, Japan) at a working distance of 10.6 mm and an operating voltage of 15 kV. Backscattered electron imaging provided high-resolution surface images of CaCO_3_ crystals, while EDS detected X-rays emitted from the sample upon electron bombardment. These X-rays revealed the chemical composition of the crystals. Multiple surface areas were analyzed to ensure accurate identification of the CaCO_3_ crystals’ elemental makeup. The X-ray spectra were analyzed using Bruker’s ESPRIT 2 software (version 2.0, Bruker Corporation, Billerica, MA, USA).

For crystal structure and phase analysis, the soil samples were dried at room temperature, pulverized into fine powder, and homogenized using a ball mill. The powdered samples were then pelletized using a hydraulic press for uniformity. X-ray diffraction (XRD) analysis was performed on an Empyrean device, utilizing Cu-Kα radiation (1.5406 Å) to identify the crystalline phases of CaCO_3_. X’Pert HighScore Plus software (version 5.3, Malvern Panalytical B.V., Almelo, The Netherlands), compared diffraction patterns with those in the International Centre for Diffraction Data (ICDD) database.

Fourier-transform infrared (FTIR) spectroscopy was employed to analyze the crystal morphology of the CaCO_3_. Pellets were prepared by mixing the soil sample with potassium bromide (KBr), which were then analyzed using an IRAffinity-1S spectrometer (Shimadzu Corporation City, Kyoto-Shi, Japan) with a spectral range of 400–4000 cm^−1^.

### 2.9. Thermal Analysis

Thermogravimetric analysis (TGA) and differential scanning calorimetry (DSC) were used to evaluate the thermal properties of MICP-treated soil. TGA was performed using a TGA 8000 instrument (PerkinElmer, Waltham, MA, USA) on 20 mg samples, heating from 30 °C to 600 °C at 10 °C/min under high-purity nitrogen. DSC was conducted with a DSC 8000 (PerkinElmer, Waltham, USA) on 5 mg samples, using an aluminum dish and heating from 30 °C to 600 °C at 10 °C/min in a nitrogen environment with a 30 mL/min flow rate. Both analyses used calibrated systems and reference materials to ensure accuracy. The durability of MICP-treated soil was assessed through wet–dry and freeze–thaw cycles. Soil columns were submerged in 3 L of distilled water for 12 h, then dried at 60 ± 2 °C for 12 h, repeating the cycle three times. For freeze–thaw testing, columns were frozen at −20 ± 2 °C for 12 h and thawed at 24 ± 2 °C for 12 h, also repeating this cycle three times. The mass loss of biocemented soil after these cycles was measured to evaluate durability. Three cycles were chosen to represent accelerated environmental testing conditions, simulating real-world exposure to moisture and freezing conditions. The columns were weighed before and after each cycle to measure mass loss, allowing for the evaluation of biocementation durability.

### 2.10. Durability Test Using Enriched Cultures

Wet–dry and freeze–thaw studies were conducted to assess the durability of soil treated with ureolytic bacterial consortium by MICP. Samples of biocemented soil that have undergone biocementation testing were utilized. In the wet–dry cycle, soil columns were submerged in 3 L of distilled water for 12 h, and after that, they were dried for an additional 12 h at 60 ± 2 °C in an oven. Afterward, three cycles of this procedure were carried out. To simulate winter and summer circumstances, soil columns were frozen (−20 ± 2 °C) for 12 h and then thawed at ambient temperature (24 ± 2 °C) for an additional 12 h. The samples were thoroughly soaked for three hours by immersion in distilled water before testing. There were three iterations of this cycle as well. All experiments were conducted in triplicate, and the biocemented soil’s mass loss after both wet–dry and freeze–thaw cycles was measured to determine the durability of MICP-treated soil.

### 2.11. Statistical Analysis

The experiments were conducted in triplicate, and the average results were obtained. Statistical analyses were performed, including ANOVA alongside post hoc analysis with the Tukey test. GraphPad Prism^®^ (version 10.2.3, GraphPad Software Inc., San Diego, CA, USA) was used for data analysis and figure plotting.

## 3. Results and Discussion

### 3.1. Biostimulation of Native Ureolytic Microbes

**Figure 1:**
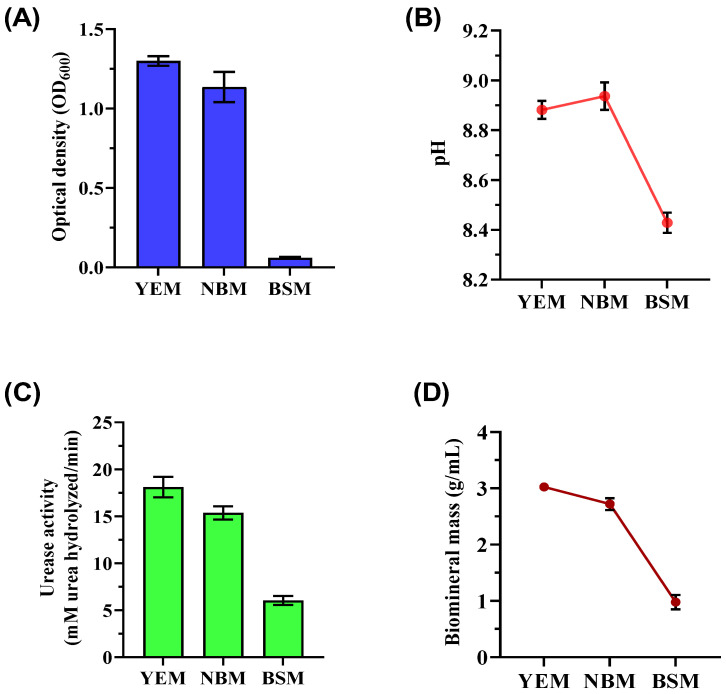
Biostimulation of ureolytic bacterial cultures was used for biomineralization after being grown in different media (YEM, NBM, and BSM) for 72 h of incubation at 32 °C with shaking at 150 rpm. The figure includes (**A**) biomass measurements (OD_600_), (**B**) pH levels, (**C**) urease activity of the bacteria, and (**D**) mass of CaCO_3_ precipitates following biomineralization tests.

This study focused on enriching ureolytic microorganisms capable of precipitation of CaCO_3_ through selective media (YEM, NBM, and BSM). Key findings demonstrated significant variations in bacterial growth, urease activity, and biomineralization efficiency depending on the medium composition, providing insights into optimizing MICP. Optical density (OD_600_) measurements revealed that the YEM culture supported the highest bacterial biomass (OD_600_ of 1.300 ± 0.033), highlighting yeast extract as a crucial nutrient source promoting consistent bacterial growth (Figure 1A). In contrast, the NBM culture displayed moderate growth (OD_600_ of 1.136 ± 0.092) with greater variability. The BSM culture, containing brown sugar without additional nutrients, produced the least biomass (OD_600_ of 0.060 ± 0.005), underscoring the inadequacy of brown sugar alone to sustain robust bacterial growth. These results align with previous findings emphasizing the importance of amino acids, vitamins, and other organic compounds for ureolytic bacterial metabolism [36]. The pH measurements of the enriched bacterial culture varied depending on the medium (Figure 1B). The end pH of the YEM culture was 8.88 ± 0.03, which is much higher than the beginning pH. This increase is probably the result of metabolic activity producing alkaline products. The NBM culture attained a comparable pH of 8.93 ± 0.05 but with more fluctuation. The BSM culture exhibited reduced metabolic activity, which was evidenced by its pH of 8.43 ± 0.04.

Urease activity varied significantly among cultures, directly influencing CaCO_3_ precipitation (Figure 1C). The YEM culture exhibited the highest urease activity (18.11 ± 0.99 mM urea hydrolyzed/min), correlating with its superior biomass production and the availability of rich nutrients. The NBM culture demonstrated moderate urease activity (15.36 ± 0.73 mM urea hydrolyzed/min), while the BSM culture showed the lowest activity (5.87 ± 0.54 mM urea hydrolyzed/min). Ureolytic bacteria raise the microenvironment’s pH through urea decomposition, facilitating CaCO_3_ precipitation [37]. The mass of CaCO_3_ precipitates correlated closely with bacterial growth and urease activity (Figure 1D). YEM culture produced the highest precipitates (3.02 ± 0.07 g/mL), which was attributed to optimal bacterial growth and enzymatic efficiency. Comparatively, NBM yielded slightly lower precipitates (2.72 ± 0.10 g/mL), while the BSM culture generated the least precipitates (0.98 ± 0.10 g/mL). Following biomineralization, the samples from YEM and NBM maintained relatively steady pH levels (YEM: 7.93 ± 0.12, NBM: 7.85 ± 0.18), but the BSM culture had a slightly lower average pH of 7.22 ± 0.18.

**Figure 2:**
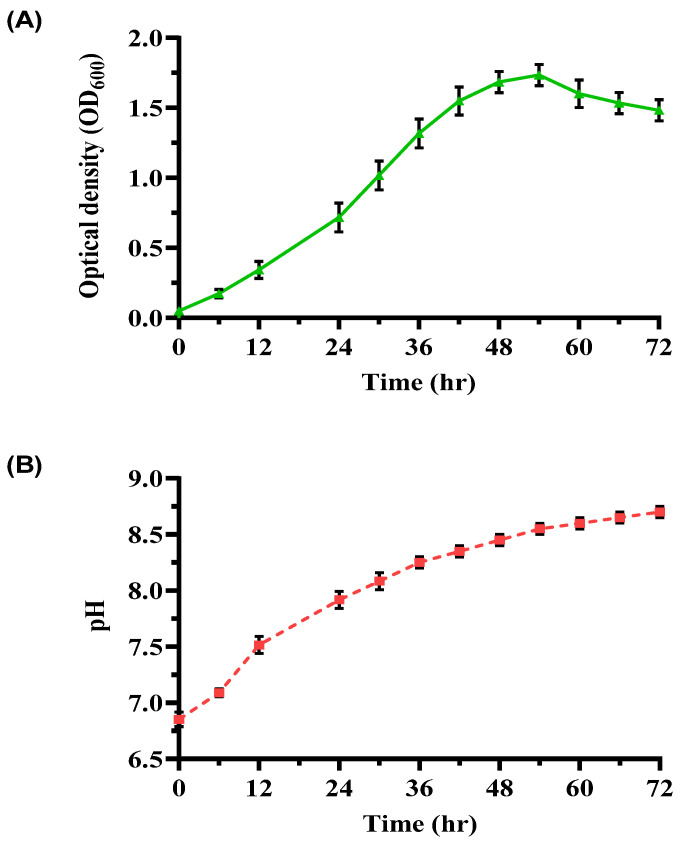
Growth and pH profile of ureolytic bacterial culture in YEM medium. (**A**) OD_600_ of bacterial cultures over 72 h, and (**B**) pH profile of the enriched bacterial culture over the same period. Error bars represent standard deviations.

An investigation into bacterial growth and pH dynamics in a YEM medium over 72 h (Figure 2) provided further insights. The growth curve exhibited distinct phases: a lag phase (0–6 h) with slow adaptation, an exponential phase (6–48 h) marked by rapid cell division (OD_600_ peaked at 1.75 ± 0.05), and a stationary phase (48–54 h) where growth plateaued due to nutrient depletion. Subsequently, OD_600_ declined to 1.48 ± 0.10 during the decline phase (54–72 h). The pH increased steadily from 6.85 ± 0.07 to 8.70 ± 0.05, signifying efficient urease activity and alkaline by-product formation. The observed growth rate (0.068 h^−1^) and doubling time (10.21 h) underscore the suitability of YEM for cultivating ureolytic bacteria with high metabolic activity.

These findings highlight the critical influence of medium composition on the growth, metabolism, and biomineralization potential of ureolytic bacteria. Rich nutrient sources like yeast extract enhance bacterial proliferation and improve enzymatic efficiency, making YEM a superior choice for MICP applications. The results suggest that future research should explore medium optimization to balance cost and efficiency for large-scale biomineralization. Additionally, investigating the role of supplementary nutrients in carbon-source-limited media like BSM could further improve the scalability of MICP for environmental and industrial applications. To maximize the effectiveness of MICP and scale up the procedure for real-world applications, it is crucial to consider how the culture medium composition affects bacterial growth and metabolism [28,38,39]. MICP efficiency and microbial growth can be hindered when a single carbon source, such as sucrose (in BSM), is used without providing other necessary nutrients [36]. Ureolytic bacteria require specific nutrients, such as amino acids, vitamins, and organic substances, for optimal development and enzyme performance. A selective culture medium incorporates inhibitors to isolate particular bacterial species and eliminate undesirable microorganisms.

### 3.2. Ureolytic Bacterial Community in Biostimulated Leachate

**Figure 3:**
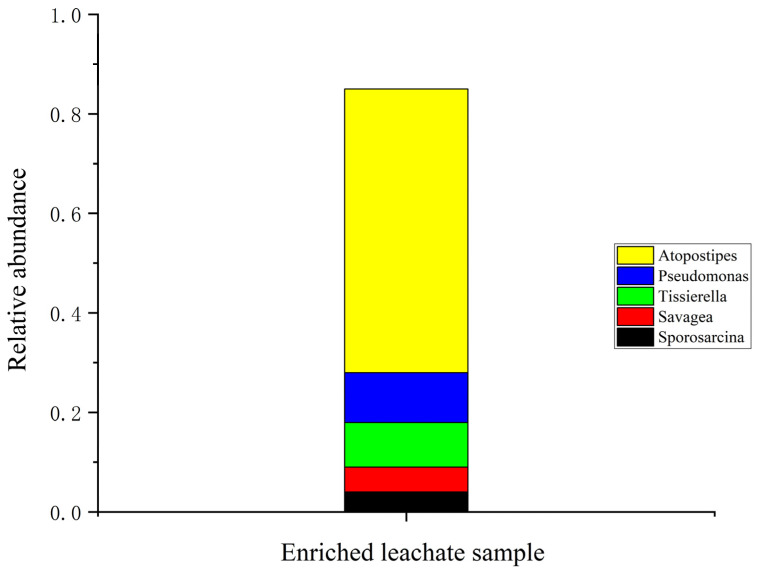
Genus abundance observed in the enriched leachate.

The bacterial community analysis of the ureolytic bacterial community in biostimulated leachate revealed Firmicutes as the dominant phylum, comprising over 80% of the bacterial population (Figure S1). This highlights its critical role in nitrogen cycling and ureolytic processes. Smaller proportions of other phyla, including Proteobacteria and Actinobacteria, were also identified. At the class level, Bacilli, Clostridia, and Gammaproteobacteria were the most abundant with Bacilli dominating due to its well-documented ureolytic activity. At finer taxonomic levels (Figure S2), the order Bacillales and Lactobacillales were key contributors to urea hydrolysis (Figure S3). The genus *Atopostipes* was the most dominant (Figure 3), playing a significant role in urea degradation, while supporting genera such as *Pseudomonas* and *Tissierella* also contributed [15,40].

These findings align with previous studies such as Marín et al. [41] and Liu et al. [42], identifying similar microbial communities dominated by *Bacillus* and *Sporosarcina*. Additionally, Oshiki et al. [43] and Hu et al. [44] emphasized the significance of *Proteobacteria*, *Firmicutes*, and *Actinobacteria* in nitrogen cycling and their resilience in challenging environments. Synergistic interactions among *Bacillus* and *Pseudomonas* species could further enhance biomineralization efficiency. One limitation of this study is the lack of bacterial community data before biostimulation. Future research will include metagenomic analysis to better understand microbial dynamics and optimize MICP applications. Enriched bacterial communities represent a cost-effective approach for bioremediation and soil stabilization, especially in wastewater-abundant regions like Malaysia.

### 3.3. Impact of pH and Temperature on Enriched Ureolytic Culture

**Figure 4:**
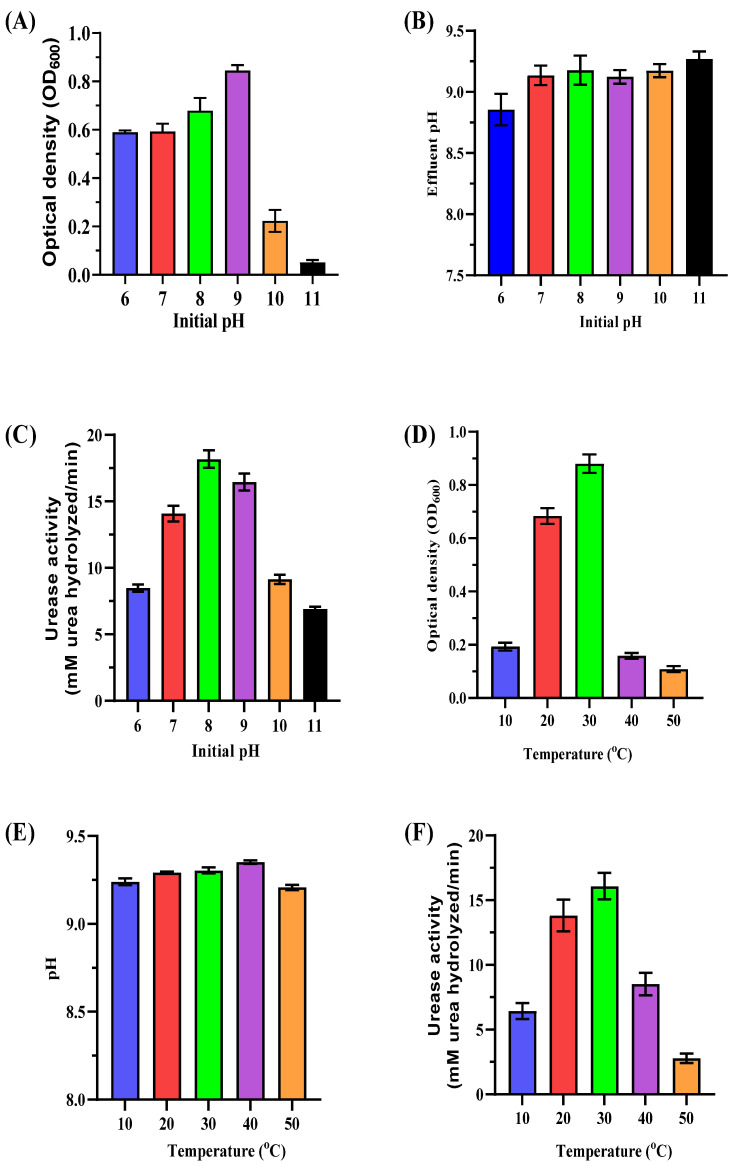
Impact of initial pH and temperature on enriched ureolytic cultures. (**A**–**C**) Effect of initial pH levels (6–11): (**A**) biomass measurements (OD_600_) of the cultures; (**B**) pH measurements of the cultures; (**C**) urease activity (urea hydrolyzed/min). (**D**–**F**) Effect of temperature (10–50 °C): (**D**) biomass measurements (OD_600_) of the cultures; (**E**) pH measurements of the cultures; (**F**) urease activity (urea hydrolyzed/min). Error bars represent standard deviations.

Optimizing MICP requires understanding how pH and temperature impact the growth and urease activity of ureolytic bacteria. This study focuses on these parameters to maximize efficiency in biostimulated systems using native bacterial consortia, reducing reliance on non-native species. The initial pH of the medium significantly affected bacterial biomass and urease activity (Figure 4A–C). Maximum biomass (OD_600_ of 0.86 ± 0.02) was observed at pH 9, while lower pH values (6–8) also supported growth with moderate biomass levels (OD_600_ ranging from 0.59 ± 0.01 to 0.68 ± 0.05). Higher pH levels (10–11) reduced biomass (OD_600_ of 0.22 ± 0.05 and 0.05 ± 0.01, respectively). Urease activity peaked at pH 8 (18.97 ± 0.73 mM urea hydrolyzed/min) with significant activity maintained at pH 9 (16.83 ± 0.55 mM urea hydrolyzed/min). Alkalinity increased post-incubation with final pH values stabilizing between 8.86 ± 0.12 and 9.27 ± 0.10 across all tested initial pH levels. These results highlight pH 8–9 as the optimal range for bacterial growth and urease activity, which is crucial for effective CaCO_3_ precipitation. Maintaining this range enhances carbonate ion production and promotes efficient biomineralization.

The initial pH of the medium influences the bacterial community composition with certain ureolytic bacteria thriving at specific pH ranges [41]. An alkaline initial pH is optimal for urease activity in MICP, reducing contamination risk and enhancing urease production. Studies show that pH increases from 6.5 to 8.5 initially inhibit some bacterial growth but eventually boost CaCO_3_ production by 25% as bacteria acclimate [45,46]. Ammonia release and fixation are also pH-dependent with optimal fixation at pH 5. Higher pH is preferred for urea hydrolysis compared to acidic conditions. In geopolymer concrete, increasing pH improves ureolytic bacterial performance [47]. While high pH enhances urease activity, excessively high levels can reduce effectiveness. *Sporosarcina* sp. and *Sporosarcina pasteurii* exhibit improved urease activities up to pH 12.5 with daily media renewal, but optimal conditions are generally below extreme pH levels [48]. This highlights the need for appropriate pH conditions to optimize ureolytic bacteria for leachate treatment and biocementation applications.

Temperature significantly influenced bacterial biomass and urease activity (Figure 4D–F). Maximum biomass (OD_600_ of 0.88 ± 0.03) and urease activity (16.94 ± 1.001 mM urea hydrolyzed/min) occurred at 30 °C. Moderate biomass and activity levels were observed at 20 °C (OD_600_ of 0.68 ± 0.03, urease activity of 13.73 ± 1.11 mM urea hydrolyzed/min). Both parameters declined sharply at lower (10 °C) and higher temperatures (40–50 °C). At 50 °C, urease activity dropped to 2.71 ± 0.32 mM urea hydrolyzed/min, indicating excessive heat denatures enzymes and inhibits bacterial growth. The results demonstrate that pH 8–9 and 25–30 °C temperatures are optimal for MICP processes. These conditions enhance bacterial metabolism, urease activity, and carbonate precipitation while minimizing extreme pH or temperature inhibitory effects.

Native ureolytic bacteria such as *Sporosarcina pasteurii* thrive in these conditions, offering practical biocementation and leachate treatment solutions. However, excessively high pH or temperature reduces efficiency, underscoring the need for precise environmental control. Temperature significantly affects the efficacy of ureolytic cultures in MICP. The optimal range for maximizing urease activity and calcite precipitation is 25 °C to 30 °C [49]. Bacterial growth and enzyme activity are enhanced within this range; temperatures below 20 °C or above 35 °C inhibit these processes [17,49]. At 35 °C, protein denaturation and cell death can reduce mineralization [50]. *Sporosarcina pasteurii* shows low urease activity below 15 °C, making it less effective in MICP at lower temperatures [17]. While urease activity peaks at 70 °C, such high temperatures are impractical for field use. Research indicates urease activity drops below 16 °C, potentially halting calcium carbonate precipitation at around 10 °C [51]. *Bacillus megaterium* has more stable urease activity across a broader temperature range, making it effective at lower temperatures [52]. A temperature of 30 °C is optimal for calcite precipitation in *S. saprophyticus* and *S. pasteurii*, but efficiency decreases at 50 °C [53]. Temperature also impacts the integration of elements like magnesium into carbonate minerals, affecting the long-term stability and utility of MICP-treated materials [54,55].

### 3.4. Preliminary Findings on Heavy Metal Immobilization

**Figure 5:**
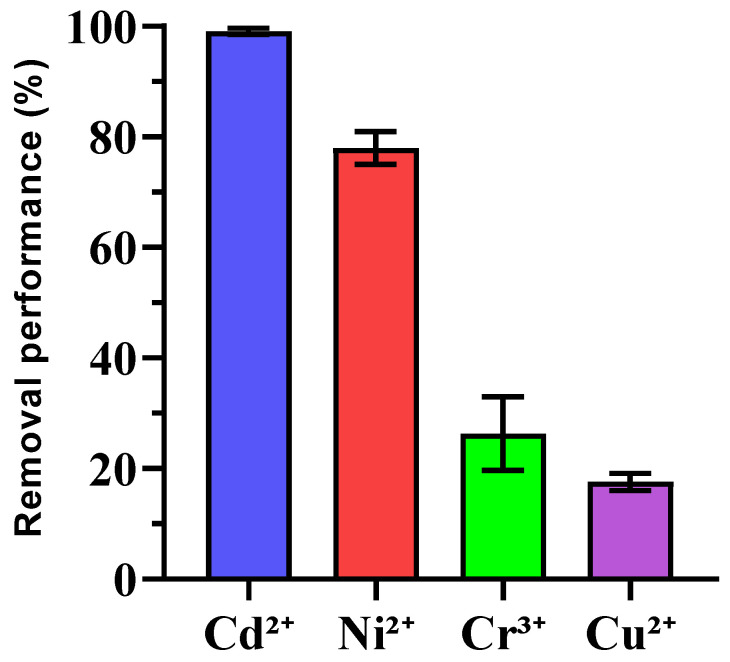
Removal percentage of heavy metal ions using enriched ureolytic bacterial culture from leachate. Error bars represent standard deviations.

The removal efficiencies of heavy metals (Cu^2+^, Cd^2+^, Cr^3+^, and Ni^2+^) by enriched ureolytic bacterial consortia were examined, as shown in Figure 5. Cd^2+^ exhibited very high removal efficiency (99.10% ± 0.53), showing strong bioremediation potential, which was consistent with other studies where ureolytic bacteria immobilize cadmium via calcium carbonate precipitation [56,57]. Ni^2+^ also had significant removal (76.33% ± 2.94), although its variability suggests the need for condition optimization, like adjusting pH or metal concentrations [58]. Cr^3+^ removal was moderate (26.67% ± 6.73), and further research is needed to enhance its immobilization with factors such as competing ions and pH playing roles. In contrast, Cu^2+^ had the lowest removal efficiency (17.61% ± 1.36), which was likely due to copper’s complexation behavior and toxicity to bacteria. Future work may focus on improving Cu^2+^ removal using copper-tolerant bacteria or mixed microbial communities to mitigate copper toxicity. Statistical analysis revealed significant differences between the metals with Cd^2+^ and Ni^2+^ outperforming Cr^3+^ and Cu^2+^. No significant difference between Cr^3+^ and Cu^2+^ was noted, indicating similar challenges for these metals.

Further studies will focus on determining the minimum inhibitory concentration of enriched cultures to better understand their tolerance to heavy metals. Spectroscopic and microscopic techniques will help characterize metal immobilization and interactions with calcium carbonate precipitation. Exploring indigenous ureolytic consortia from leachate wastewater could provide a low-cost alternative for heavy metal removal, leveraging the microbial communities’ complementary metabolic capabilities [59]. Addressing bacterial sensitivity to toxic ions and metal complex formation will be essential for improving bioremediation. Optimizing pH and temperature and using bacterial consortia could enhance heavy metal immobilization, leading to better environmental outcomes. These strategies are crucial for scaling up bioremediation technologies for practical applications in contaminated wastewater and soil.

### 3.5. Crystal Structure and Mineralogical Composition of Treated Soil

**Figure 6:**
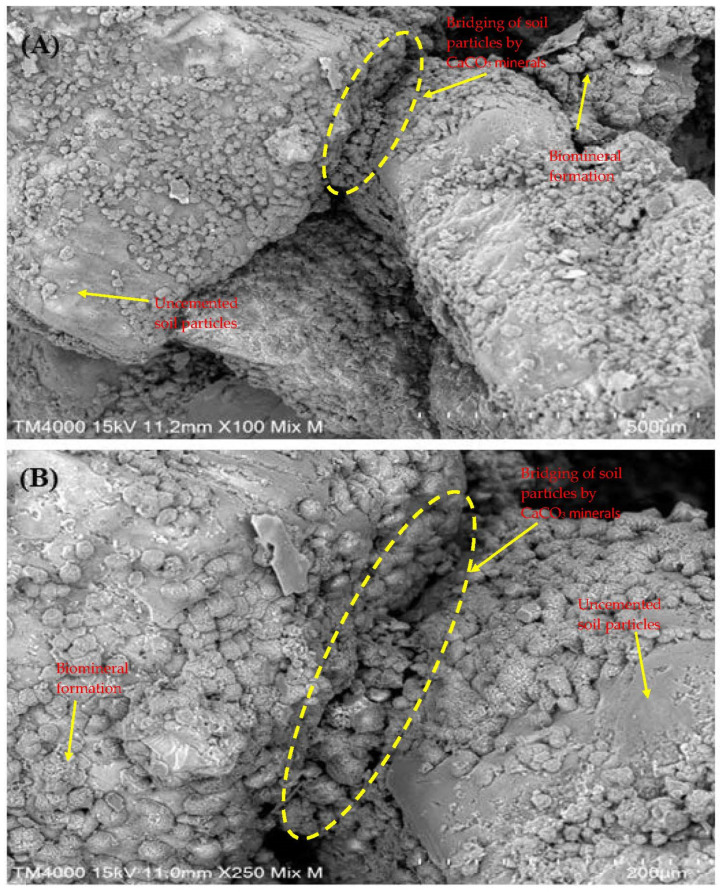
Crystal morphology of MICP-treated soil revealing well-defined rhombohedral and polyhedral crystal forms bridging soil particles and filling voids within the soil matrix. (**A**) SEM image at ×100 magnification; and (**B**) SEM image at ×250 magnification.

The SEM images in Figure 6 illustrate the crystal morphology and distribution on soil specimens treated with MICP using enhanced native ureolytic bacterial consortium. At lower magnification (×100, Figure 6A), minute, scattered particles form a uniform coating on soil surfaces with some clustered regions indicating active bacterial calcite precipitation. At higher magnification (×250, Figure 6B), crystals exhibit distinct rhombohedral and polyhedral shapes typical of calcite, bridging soil particles and filling voids crucial for stabilization. The crystals, with an average length of 21.91 ± 7.18 µm and area of 19.37 ± 6.78 µm^2^, demonstrate high bacterial activity and effective calcite precipitation, enhancing soil strength and reducing porosity. The uniform distribution and well-defined shapes affirm the efficacy of MICP treatment. Crystal bridging, which strengthens soil and decreases permeability, highlights MICP’s potential for construction and geotechnical applications. This study showcases how ureolytic bacteria from landfill leachate can facilitate in situ bioremediation, immobilizing contaminants and mitigating environmental impacts. The results emphasize MICP as an environmentally friendly approach to improving soil properties and reducing contamination. The crystals, which have different sizes and rhombohedral and polyhedral forms, cover the soil particles and act as a bridge between them, filling in spaces and strengthening the cohesiveness of the soil. As noted, crystal bridging is critical in strengthening soil and decreasing permeability, making it a potentially viable material for construction and geotechnical applications [60]. Utilizing ureolytic bacteria from landfill leachate shows how natural bacterial communities may be used for in situ bioremediation, successfully immobilizing contaminants and lessening their adverse environmental effects [30,61].

**Figure 7:**
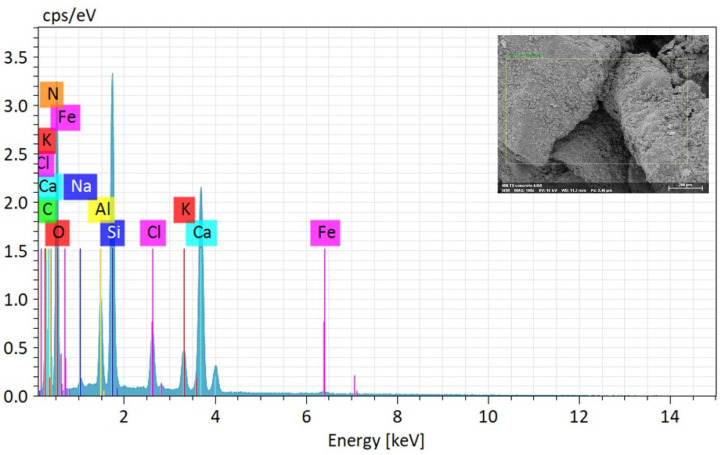
Elemental composition of MICP-treated soil via EDS analysis. The yellow rectangular lines indicate the specific locations where the EDS analysis was performed on the soil samples.

The elemental composition of MICP-treated soil employing enhanced ureolytic bacterial cultures from landfill leachate wastewater is fundamentally understood from the EDS study shown in Table 2 and Figure 7. The following SEM picture identifies the EDS analysis point position on the soil samples by faint yellow rectangular lines. Numerous elements, such as calcium (Ca), silicon (Si), aluminum (Al), iron (Fe), potassium (K), chlorine (Cl), sodium (Na), carbon (C), oxygen (O), and nitrogen (N), are shown by the EDS spectrum. Of these, the components carbon (C) and calcium (Ca) show pronounced peaks, indicating that the precipitation of CaCO_3_ within the soil matrix has occurred successfully [62,63]. The quantitative elemental composition of biominerals (EDS) on soil specimens following biocement treatment with enhanced ureolytic bacterial cultures is shown in Table 2. O is the most prevalent element with an identified abundance of 49.87% by mass and 58.76% by atom. Ca, Si, and C are next in importance. The high carbon content confirms carbonate production, the presence of silica (SiO_2_) and CaCO_3_ crystals shows effective biocementation, and the presence of N indicates the existence of organic matter or ammonium molecules, most likely from urea hydrolysis [59]. Impurities such as Al, Cl, K, and Fe are trace elements that add to the treated soil’s resilience. The noteworthy percentages of SiO_2_ (10.54%) and Ca (16.35%) are essential for stabilizing the soil and improving structural integrity. The leachate’s Cl, K, and Na are integrated into the biomineral structure, which may impact the soil’s stability and mechanical characteristics. Although it raises questions regarding microbial activity, the minor amount of Fe may improve durability. The sustainable method of using landfill leachate for MICP reduces environmental effects while stabilizing soil by using its nutrients to increase microbial activity and calcite precipitation.

**Figure 8:**
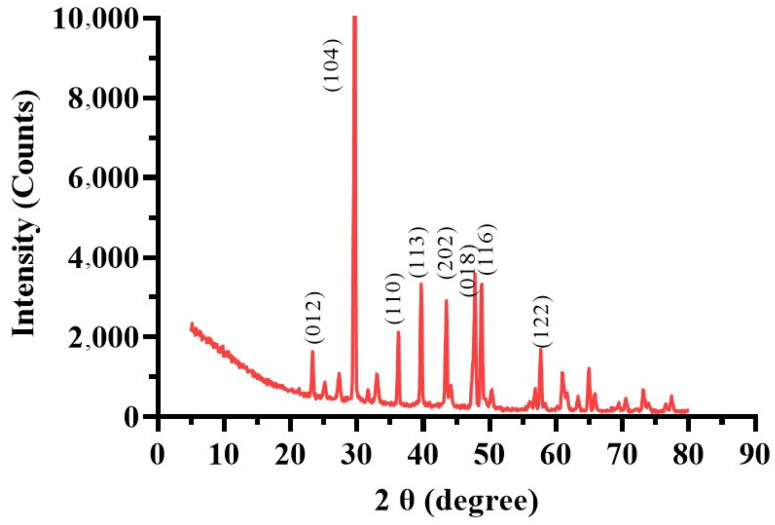
X-ray diffraction (XRD) pattern of the MICP-treated soil sample.

XRD analysis (Figure 8) identified calcite in the MICP-treated soil with diffraction peaks matching ICDD database card 00-047-1743. Peaks at 012, 104, 110, 113, 202, 018, 116, and 122 degrees 2θ indicated the predominance of calcite. The highest intensity peak (13,420.61 counts at 29.6711° 2θ) demonstrated a dominant crystalline phase essential for enhancing soil stability by strengthening mechanical properties, reducing permeability, and binding soil particles [64]. The peaks’ high-intensity counts (10,000) suggest that the calcite structure’s (104) crystallographic plane has a comparatively high abundance. In addition to the prominent calcite peaks, the XRD analysis also showed a few low-intensity peaks. For example, at 34.8651° 2θ, a peak with the lowest intensity of 310.6655 counts was seen. This suggests that the sample contains smaller or perhaps interacting crystalline components. There were also low-intensity peaks found at 21.3452° 2θ with 450.1234 counts and at 38.5678° 2θ with 289.4567 counts. The complex composition of the landfill leachate wastewater used for bacterial biostimulation, which always comprises a variety of organic and inorganic chemicals, may cause contaminants [65].

**Figure 9:**
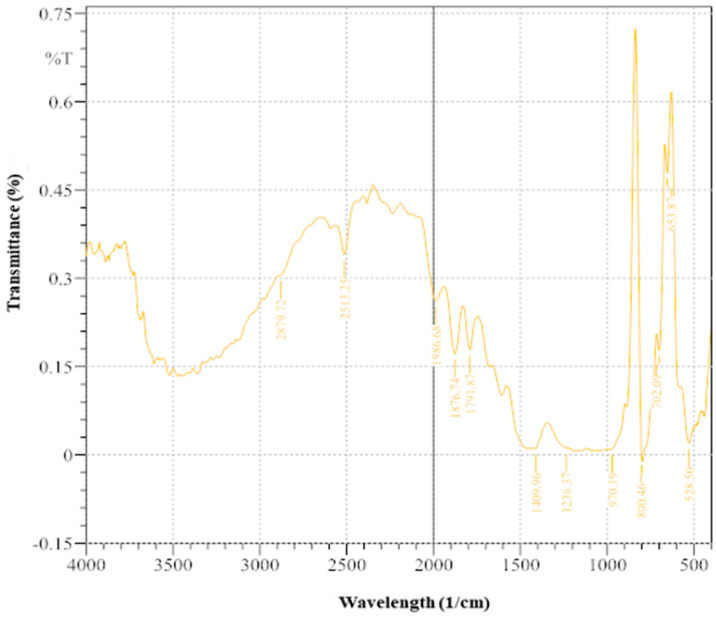
FTIR image of soil sample after treatment with enriched ureolytic bacterial cultures.

The FTIR spectra (Figure 9) provide insights into the implications of MICP using ureolytic bacteria enriched from wastewater leachate. Significant ramifications exist for the various functional groups and chemical elements in the treated soil [12]. A low-intensity peak at 528.5 cm^−1^ (0.019997) indicates minimal metal–oxygen bonds or metal oxides. The moderate intensity at 653.87 cm^−1^ (0.479136) suggests metal–oxygen connections or metal-carbonate interactions, indicating interactions with precipitated calcite. The peak at 702.09 cm^−1^ (0.177542) suggests minor functional groups. The absence of significant intensity at 800.46 cm^−3^ and low intensity at 970.19 cm^−1^ (0.010502) indicate trace components or weakly bound functional groups. Peaks at 1236.37 cm^−1^ (0.011323) and 1409.96 cm^−3^ (0.01058) suggest poorly bound carbonate groups. The peak at 1791.87 cm^−1^ (0.179111) implies the presence of organic compounds or C=O stretching vibrations. At 1876.74 cm^−1^ (0.171481), there are mild C=O stretching vibrations. A significant peak at 1986.68 cm^−1^ (0.261371) confirms the presence of carbonate groups, supporting the successful precipitation of calcite. The XRD and FTIR results together emphasize the dual role of MICP in immobilizing pollutants and stabilizing soil by integrating organic pollutants into the soil matrix [29,66].

**Figure 10:**
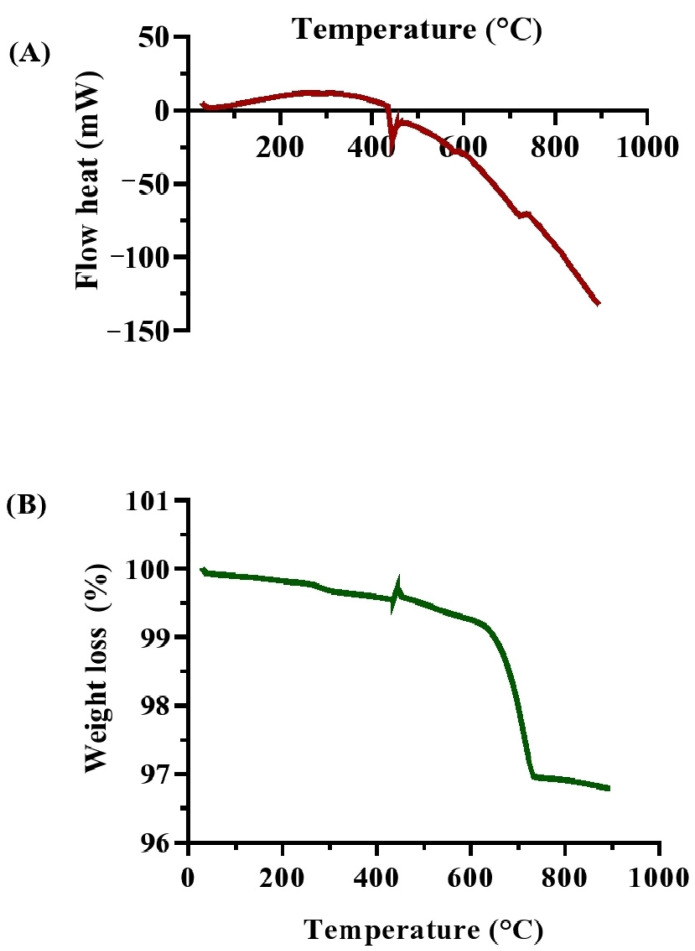
Thermal characterizations of biocemented soil samples after treatment with enriched ureolytic cultures from the leachate wastewater sample. (**A**) DSC analysis; and (**B**) TGA analysis.

The thermal studies (DSC and TGA) provide vital information on the effectiveness of biocementation treatments. DSC analysis (Figure 10A) of the soil specimens treated with MICP showed several endothermic and exothermic peaks, indicating multiple thermal events. The DSC signal started at about 30 °C (4.90803 mW) and dropped to a local minimum at 48 °C (2.05271 mW), indicating the loss of physically adsorbed water. The signal increased between 75 °C and 120 °C (5.02854 mW), which was likely due to the release of bound water and the beginning of CaCO_3_ phase dehydration. A significant endothermic peak at around 300 °C (11.6293 mW) suggested the breakdown of organic molecules. The signal remained steady until 318 °C before decreasing rapidly, reaching an exothermic transition at 444 °C (−20.702 mW), representing the thermal decomposition of CaCO_3_ into CO_2_ and CaO [67]. This peak confirms the successful production of CaCO_3_, enhancing soil stability.

The DSC readings continued to decrease until 894 °C (−132.398 mW), consistent with the gradual breakdown of carbonate phases, demonstrating the MICP process’s efficiency in producing stable carbonate minerals [68]. TGA analysis (Figure 10B) detailed the weight loss profile of the MICP-treated soil as a function of temperature. The initial weight dropped from 99.9973% to 99.881% between 30 °C and 120 °C, indicating the loss of physically adsorbed moisture. Between 120 °C and 330 °C, the weight reduced to 99.6514%, reflecting the soil matrix’s hydration. A noticeable weight loss occurred between 330 °C and 723 °C, with the weight falling to 96.9741%, including early phases of CaCO_3_ decomposition and the breakdown of organic substances. The most significant weight loss, from 96.9741% to 96.7892%, happened between 723 °C and 894 °C, which was consistent with the thermal breakdown of CaCO_3_ into CaO and CO_2_ [11]. The steady weight loss indicates the presence of calcite in the soil matrix, confirming the effectiveness of MICP treatment in soil stabilization [68].

### 3.6. Strength, CaCO_3_ Contents, and Monitoring of Effluent

**Figure 11:**
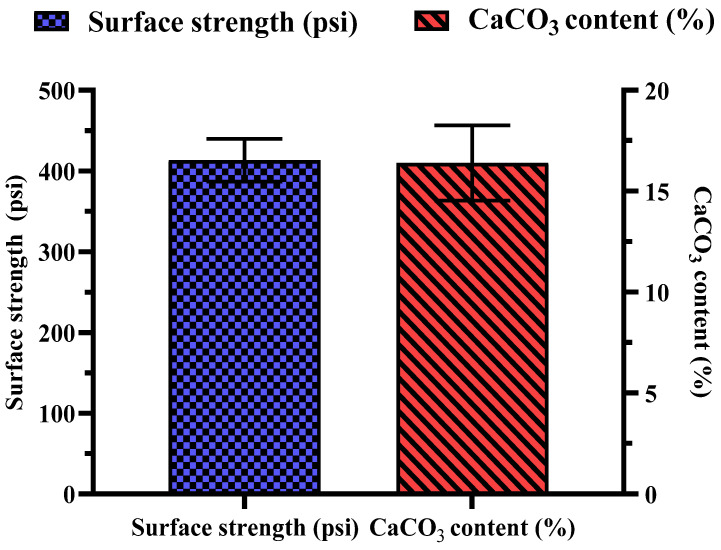
Measurement of MICP treatment showing surface strength and CaCO_3_ content. Error bars represent standard deviations.

**Figure 12:**
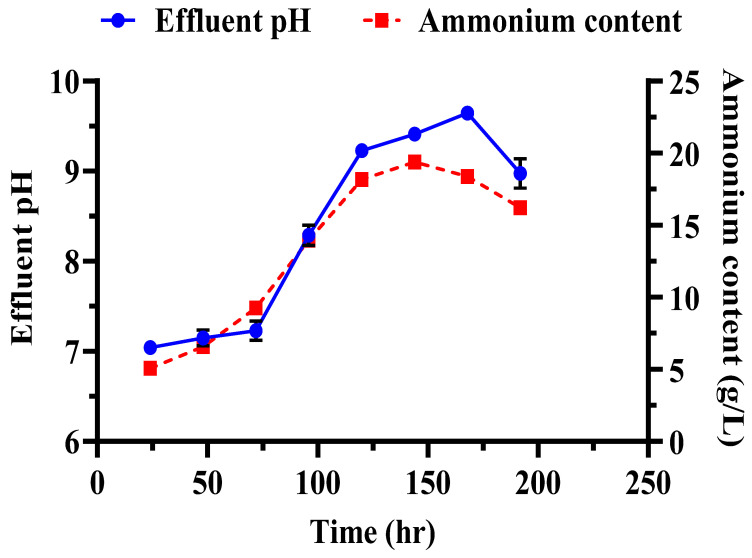
Evaluation of effluents during soil biocementation with enriched ureolytic cultures. The pH levels and ammonium concentrations in the effluent samples collected throughout the treatment process are shown. Error bars denote the standard deviations.

Several key observations were made during the biocementation treatment of the sandy soil column with enhanced ureolytic cultures. A pungent odor, likely from ammonia, indicated active bacterial activity [13]. White precipitates appeared on the soil surface on the second day, indicating CaCO_3_ formation. The MICP treatment significantly improved soil stability and CaCO_3_ content with the mean surface strength and CaCO_3_ content at 413.33 ± 26.11 psi and 16.73 ± 1.86%, respectively (Figure 11). The highest surface strength (440 psi) correlated with the highest CaCO_3_ content (18.83%), highlighting the role of calcite in enhancing soil mechanical properties [48,50,69]. Indigenous ureolytic microorganisms performed better than commercial strains, particularly at lower temperatures (15 °C), due to longer bacterial retention and steady enzyme activity [19,70,71]. This natural adaptation reduces costs and supports sustainable soil stabilization and cleanup efforts [72]. Monitoring effluent pH and ammonium concentration during biocementation (Figure 12) revealed significant trends. The effluent pH progressively increased from an average of 7.04 ± 0.03 at 24 h to 9.69 ± 0.09 at 192 h, indicating ongoing ureolytic activity [73]. The ammonium concentration initially rose from 5.07 ± 0.03 mg/L at 24 h to a peak of 19.42 ± 0.12 mg/L at 144 h; then, it slightly declined to 16.22 ± 0.09 mg/L at 192 h. This pattern reflects ammonia release during urea hydrolysis and subsequent microbial dynamics or nutrient availability changes. Increasing pH and ammonium concentration support effective calcite precipitation, enhancing soil stability [29]. The pH and ammonium peaks at 144 h indicate maximal ureolytic activity, aligning with optimal calcite precipitation timing [72].

### 3.7. Durability of MICP and Future Direction

Wet–dry and freeze–thaw cycles were used to simulate weathering and evaluate the resilience of soil specimens treated with MICP. The outcomes, detailed in Table 3, demonstrate how these cycles affect the mass loss of treated soil and shed light on the stability and resilience of the material. The cumulative mass loss of the soil specimens subjected to wet–dry cycles was 2.83 ± 0.48%. With initial losses of 1.24 ± 0.72% in the first cycle and 2.15 ± 0.92% in the second, the mass loss grew gradually with each cycle. This steady rise indicates that although the soil treated with MICP remains reasonably stable, the soil’s integrity is gradually weakened by frequent soaking and drying. The progressive disintegration or weakening of the soil structure due to repeated hydration and drying may be indicated by increased mass loss throughout subsequent cycles. After three cycles of the freeze–thaw process, the treated soil had a total mass loss of 3.82 ± 0.77%. In the first cycle, mass losses were 2.10 ± 0.84%; in the second cycle, they increased to 3.47 ± 0.49%. The findings suggest that the influence of freeze–thaw conditions is more significant than that of wet–dry cycles. The expansion and contraction of water inside soil pores during freezing and thawing is responsible for the more significant mass loss seen under freeze–thaw cycles. This can result in increased physical stress and possible soil structure failure. Table 3 demonstrates that soil treated with MICP has high durability; nevertheless, the effects of freeze–thaw cycles are greater than those of wet–dry cycles. This implies that environmental considerations are critical when using MICP-treated soil in practical situations. Although MICP treatment improves soil stability, more studies are necessary to increase resilience, since its long-term performance may differ depending on environmental factors [10,74]. By reducing maintenance costs, optimizing treatment methods, and confirming the efficacy of biocementation, durability testing makes MICP treatments more practical for large-scale applications and advances soil-stabilizing techniques.

## 4. Broader Implications and Future Prospects

### 4.1. Practical Implication of the Study

The findings from this study highlight significant practical implications for advancing sustainable solutions in soil stabilization and heavy metal remediation. By successfully utilizing landfill leachate as a biostimulant MICP, this work demonstrates a cost-effective and environmentally friendly approach to addressing contaminated soil challenges. The study’s emphasis on reducing reliance on synthetic chemicals and leveraging indigenous microbial communities aligns with global efforts to minimize ecological disruption. Moreover, the insights into ammonia mitigation and the stability of biocemented soils under diverse environmental conditions provide actionable pathways for scaling MICP from laboratory experiments to field applications.

These contributions underline the potential of this approach to serve as a viable alternative to traditional, resource-intensive soil remediation methods. The study also provides a valuable framework for addressing key challenges associated with the scalability of MICP processes, particularly in the context of variable and complex landfill leachate compositions. By demonstrating the potential of landfill leachate as a nutrient source for microbial biostimulation, this research not only offers an innovative use of an industrial waste product but also enhances the economic feasibility of MICP applications. Furthermore, the utilization of leachate helps in reducing landfill waste, contributing to overall waste management efforts. The experimental findings highlight strategies to mitigate environmental risks, such as ammonia accumulation, while maintaining effective soil biocementation and heavy metal immobilization. These practical insights contribute to bridging the gap between laboratory research and field implementation, offering tangible solutions for industries and environmental practitioners working toward sustainable land remediation and infrastructure development.

### 4.2. Environmental Impact of MICP

One of the key advantages of MICP is its significantly lower environmental impact compared to traditional remediation techniques. Traditional methods for soil stabilization and heavy metal remediation often involve the use of chemical agents, which can introduce pollutants into the environment and contribute to long-term ecological damage [30]. In contrast, MICP leverages naturally occurring ureolytic bacteria to precipitate CaCO_3_, which is a process that not only stabilizes soils but also immobilizes pollutants in an environmentally friendly manner [73,75,76,77]. Studies have demonstrated that MICP using indigenous bacteria is an effective, eco-friendly technology for remediating heavy metal-contaminated sites. For instance, Hu et al. [78] found that the ureolytic bacterium *Brucella intermedia* TSBOI, used in MICP, significantly reduced the bioavailability of heavy metals (Cu, Pb, and Zn) in contaminated soils. The process involved the transformation of heavy metals into carbonate-bound forms, effectively reducing their mobility and bioavailability [78]. This is a crucial environmental benefit, as it prevents heavy metals from leaching into groundwater, unlike traditional chemical methods that may inadvertently release pollutants back into the environment. Studies like those by Kasra et al. further support the environmental advantages of MICP [79], and Sugata et al. [80] have shown that MICP’s use of indigenous bacteria not only stabilizes the soil but also enhances the soil’s geotechnical properties without adding harmful chemicals. Kasra et al. [79] demonstrated that bacteria such as *Bacillus lentus* and *Staphylococcus aureus* could bioaccumulate and immobilize trace metals like Cd^2+^ and Hg^2+^ by converting them into stable carbonates, reducing the environmental risks associated with trace metal contamination. This is in stark contrast to conventional soil treatments that often rely on toxic or non-biodegradable chemicals, which can have long-lasting negative impacts on the environment.

Moreover, MICP’s ability to utilize indigenous microorganisms in their natural habitats minimizes the disruption to local ecosystems. Lorc et al. [81] highlighted that using native calcifying bacteria, such as *Alkalihalobacillus pseudofirmus*, in microbial treatments led to the formation of an organo-mineral coating, demonstrating how indigenous microbial communities can be harnessed for environmental repair with minimal ecological disruption [81]. In comparison, traditional soil treatments such as cement stabilization often require large-scale interventions and the transportation of materials, which contributes to carbon emissions and other environmental burdens. Another study by Naeimi [82] compared the environmental impacts of microbial and chemical grouting techniques, showing that biocementation via MICP required significantly lower calcium consumption and had a smaller environmental footprint, especially in terms of CO_2_ emissions, than traditional cement-based methods. This is because MICP processes operate at ambient temperatures and use naturally occurring calcium sources, reducing the energy-intensive requirements typical of chemical soil stabilization [82]. The MICP technique stands out for its environmentally sustainable approach to soil stabilization and pollutant removal. By using indigenous bacteria to bioprecipitate CaCO_3_, MICP not only reduces the use of harmful chemicals but also minimizes ecological disruption. The process’s ability to immobilize heavy metals and improve soil properties, while having a lower carbon footprint, positions it as a more environmentally friendly alternative to traditional remediation and stabilization methods.

### 4.3. Limitations and Future Directions

The integration of landfill leachate as a biostimulant for MICP offers a promising solution for sustainable soil improvement and heavy metal remediation. However, its use also introduces unique challenges that intersect with broader scalability and environmental concerns associated with MICP. These challenges merit deeper exploration to ensure effective and environmentally responsible implementation. One of the primary issues with leachate-based MICP is scalability. While the laboratory-scale applications of this approach have shown encouraging results, field-scale deployment often reveals complexities that are not immediately apparent. Research by Babakhani et al. [83] and Kahani et al. [84] highlights cost-effective alternatives, such as corn-steep liquor and whey, which significantly reduce production expenses. Hence, the variability in leachate composition, combined with site-specific factors like soil type and contaminant profiles, complicates the standardization of treatment processes. Therefore, when considering leachate as a potential nutrient source from industrial waste, it is crucial to account for its composition and other influencing factors.

Environmental constraints are another critical concern. The ureolysis-driven MICP process inherently produces ammonia, which is a by-product that can accumulate to potentially harmful levels. The use of leachate amplifies this challenge, as it already contains nitrogen compounds that contribute to ammonia production. Effective mitigation strategies, such as ammonia stripping or alternative enzymatic pathways, are essential for minimizing environmental risks. Studies like those by Gowthaman et al. [85] and Maleki-Kakelar et al. [86] suggest that using low-grade chemicals and non-sterile conditions can reduce some environmental impacts. However, these methods may also introduce variability in bacterial performance and the quality of cementation, adding another layer of complexity to field applications. Furthermore, researchers could explore utilizing little or no urea to minimize ammonia production during ureolysis, as well as developing microbial consortia with lower ammonia outputs, and optimizing reactor designs to enhance ammonia volatilization and removal efficiency.

The reliance on ureolytic bacteria from landfill leachate further complicates the MICP process. While these bacteria are naturally adapted to the leachate environment, their dynamic microbial communities can lead to variability in biocementation performance. Yan et al. [87] emphasize the importance of dominant bacterial strains for effective metal immobilization and soil improvement. Maintaining consistent microbial activity over extended periods or across diverse field conditions remains a challenge. This variability could hinder reproducibility and reduce the efficacy of large-scale MICP projects. Additionally, the long-term stability and sustainability of MICP-treated soils require further investigation. Environmental conditions, such as wet–dry cycles and freeze–thaw events, could impact the durability of biocemented soils [85]. Furthermore, introducing bacterial consortia derived from landfill leachates into the soil may have unintended ecological consequences, potentially disrupting native microbial communities. Monitoring these impacts and understanding their broader implications is crucial for ensuring the sustainability of this technology.

To address these interconnected challenges, future research must focus on optimizing leachate pretreatment processes, designing scalable and uniform application methods, and exploring alternative nutrient sources. By integrating insights from studies on food-grade yeast extract and agricultural by-products, it is possible to develop more cost-effective and reliable solutions [84,88,89]. Similarly, the development of ammonia mitigation techniques and extensive field trials will be instrumental in evaluating the practicality of leachate-based MICP under real-world conditions. Effective strategies such as adjusting the nutrient balance, utilizing advanced filtration systems, incorporating biochar, employing green amendments, and continuously monitoring and optimizing treatment processes can also help minimize environmental impacts. By addressing these limitations, MICP has the potential to transition from a promising concept to a widely adopted solution for soil stabilization and environmental remediation. Future studies could also incorporate additional controls to isolate the effects of abiotic factors, such as pH, temperature, and ionic strength, on heavy metal removal in MICP systems. By systematically varying these parameters in controlled experiments, a clearer understanding of their influence on microbial activity, carbonate precipitation, and metal immobilization could be achieved. Such investigations would strengthen causal interpretations and provide a more robust foundation for optimizing MICP-based remediation strategies under diverse environmental conditions.

## 5. Conclusions

This study explored the biostimulation of ureolytic bacterial consortium from landfill leachate using different selective media, uncovering significant variations in bacterial growth, urease activity, and biomineralization efficiency. The YEM medium emerged as the most effective, supporting high biomass and urease activity levels, leading to substantial calcium carbonate precipitation. This underscores the importance of nutrient-rich media in optimizing bacterial growth and enzymatic performance. Conversely, media lacking essential nutrients, such as BSM, exhibited limited bacterial growth and urease activity. Taxonomic analysis identified *Proteobacteria* as the dominant phylum in biostimulated leachate wastewater with *Bacillus* and *Sporosarcina* species playing crucial roles in urea hydrolysis and calcium carbonate precipitation. These findings align with previous studies, emphasizing the significance of these ureolytic bacteria in bioremediation and environmental engineering applications. The study of pH and temperature effects demonstrated that biomass production was optimal at pH 9, while urease activity peaked at pH 8. These results highlight the need for precise environmental control to maximize microbial performance and MICP efficiency.

This research successfully demonstrated the potential of MICP using indigenous ureolytic bacteria from landfill leachate as a sustainable and cost-effective method for soil stabilization. The enriched microbial consortia facilitated uniform calcite precipitation, significantly enhancing soil strength, reducing porosity, and improving the overall mechanical properties of treated soil. SEM, XRD, and EDS analyses confirmed these findings, while thermal analyses validated the stability of the precipitated calcite. A strong positive correlation between surface strength and CaCO_3_ content further illustrated the effectiveness of the MICP treatment. However, while treated soil showed resilience under wet–dry conditions, durability tests indicated greater mass loss under freeze–thaw cycles, suggesting that further optimization is needed to improve long-term durability in varying environmental conditions. In conclusion, this method offers a viable alternative to traditional chemical soil stabilization techniques and aligns with circular economy principles by utilizing waste-derived microorganisms. With further refinement, this technology has the potential to provide a scalable and environmentally sustainable solution for geotechnical engineering applications, contributing to both soil remediation and waste valorization efforts.

## Figures and Tables

**Table 1:** Compositions selected cultivation medium.

Component	YEM	NBM	BSM
Yeast extract	20 g/L	0	0
Nutrient broth	0	13 g/L	0
Brown sugar	0	0	10 g/L
Urea	40 g/L	40 g/L	40 g/L
Nickel chloride	0.02 g/L	0.02 g/L	0.02 g/L
Ammonium chloride	5 g/L	5 g/L	5 g/L
Sodium chloride	5 g/L	0	5 g/L
Initial pH	6.26	6.92	5.4

**Table 2:** Elemental compositions of biominerals after soil biocementation.

Element	Atomic Number	Mass Normalized %	Atom %
O	8	49.87	58.76
Ca	20	16.35	7.69
Si	14	10.54	7.08
C	6	10.22	16.0467
N	7	3.78	5.08
Al	13	3.00	2.10
Cl	17	3.10	1.39
K	19	2.42	1.17
Fe	26	0.75	0.27
Na	11	0.59	0.48

**Table 3:** Mass loss percentage for both wet–dry and freeze–thaw cycles.

Samples	Cycle 1 (% Loss)	Cycle 2 (% Loss)	Cycle (% Loss)
Wet–Dry	1.24 ± 0.72	2.15 ± 0.92	2.83 ± 0.48
Freeze–Thaw	2.10 ± 0.84	3.47 ± 0.49	3.82 ± 0.77

## Data Availability

The original contributions presented in the study are included in the article/Supplementary Materials; further inquiries can be directed to the corresponding authors.

## Supplementary Materials

The following supporting information can be downloaded at https://www.mdpi.com/article/10.3390/microorganisms13010174/s1, Figure S1. Phylum abundance observed in the enriched leachate sample; Figure S2. Class abundance observed in the enriched leachate sample; Figure S3. Order abundance observed in the enriched leachate sample. Figure S4. Depiction of the preparation, enrichment, and analysis processes of ureolytic bacterial cultures using different growth media, leachate samples, and various testing methods. (A) Freshly prepared media: YEM (yeast extract medium), NBM (nutrient broth medium), and BSM (brown sugar medium). (B) The leachate sample is to be inoculated into the freshly prepared media. (C) Shake flasks containing leachate and growth media placed in an incubator for the enrichment of ureolytic microbes. (D) The appearance of three different growth media after the enrichment process aimed at stimulating native ureolytic bacterial cultures. (E) Urease production assay using Christensen’s medium in universal bottles. (F) Biomineralization test of the enriched cultures in a solution containing 0.5 M urea and calcium chloride; Figure S5. The grain size distribution curve of the sand was used in this study for the biocementation test; Table S1. Physicochemical characterization of leachate sample.
