# Supplementary material for: Enhanced MICP for Soil Improvement and Heavy Metal Remediation: Insights from Landfill Leachate-Derived Ureolytic Bacterial Consortium
Source: Microorganisms. 2025 Jan 15;13(1):174. doi: 10.3390/microorganisms13010174 (PMC11767312; doi:10.3390/microorganisms13010174)
Supplement: Supplementary file 1 [file microorganisms-13-00174-s001.zip › microorganisms-3362541-supplementary.pdf]

Supplementary Data

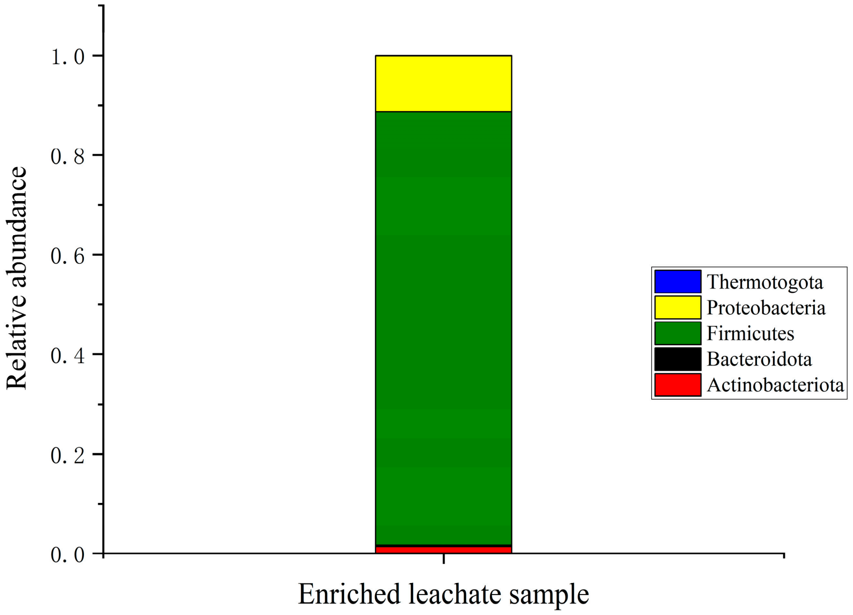

Figure S1. Phylum abundance observed in the enriched leachate sample

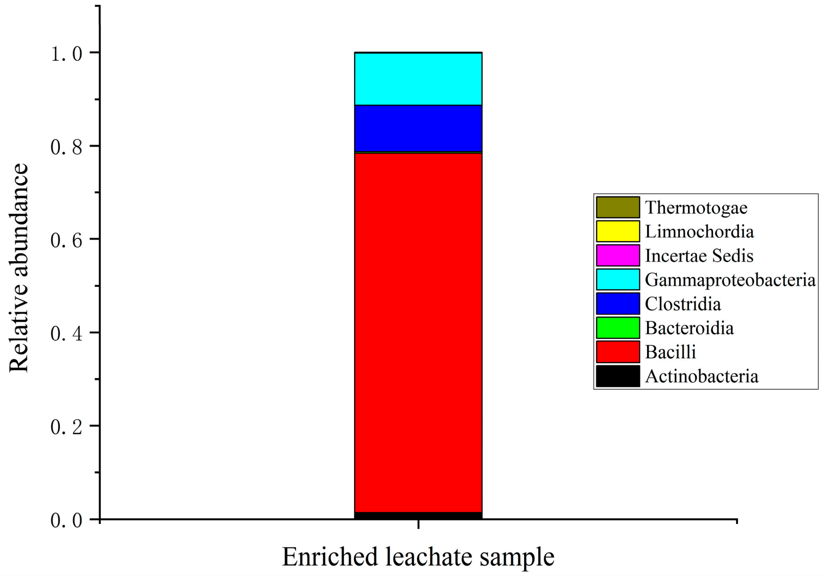

Figure S2. Class abundance observed in the enriched leachate sample

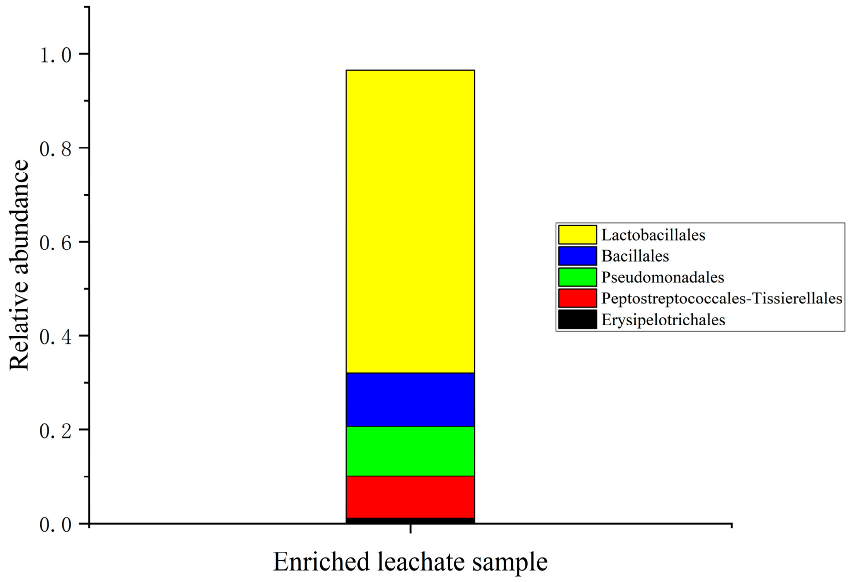

Figure S3. Order abundance observed in the enriched leachate sample

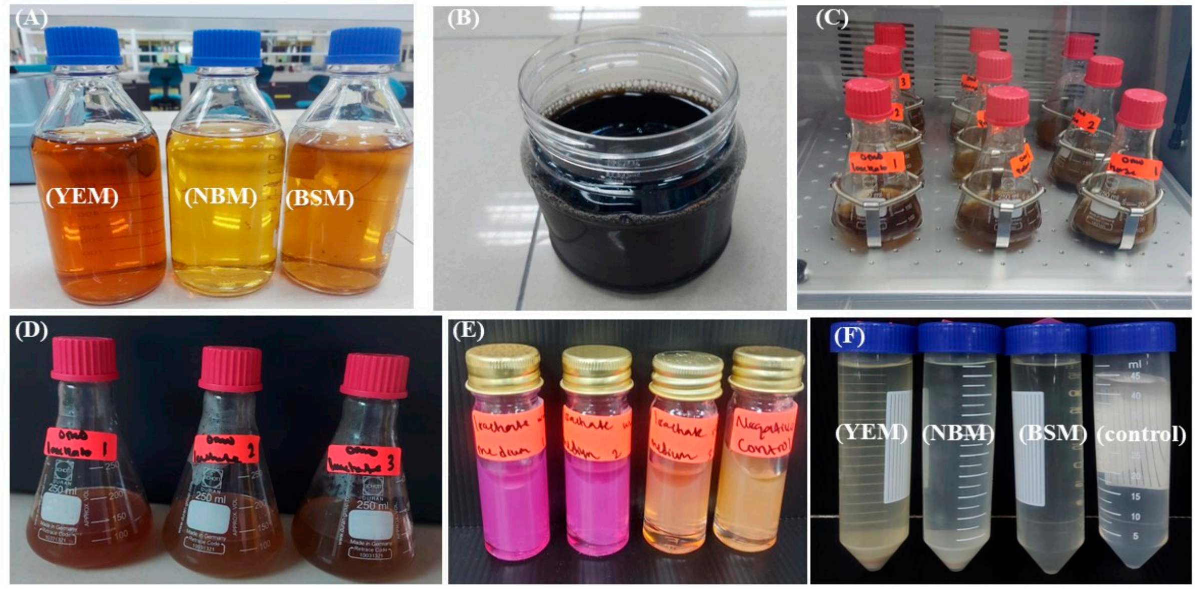

Figure S4: Depiction of the preparation, enrichment, and analysis processes of ureolytic bacterial cultures using different growth media, leachate samples, and various testing methods. (A) Freshly prepared media: YEM (Yeast Extract Medium), NBM (Nutrient Broth Medium), and BSM (Brown Sugar Medium). (B) Leachate sample to be inoculated into the freshly prepared media. (C) Shake flasks containing leachate and growth media placed in an incubator for the enrichment of ureolytic microbes. (D) The appearance of three different growth media after the enrichment process aimed at stimulating native ureolytic bacterial cultures. (E) Urease production assay using Christensen's medium in universal bottles. (F) Biomineralization test of the enriched cultures in a solution containing 0.5 M urea and calcium chloride.

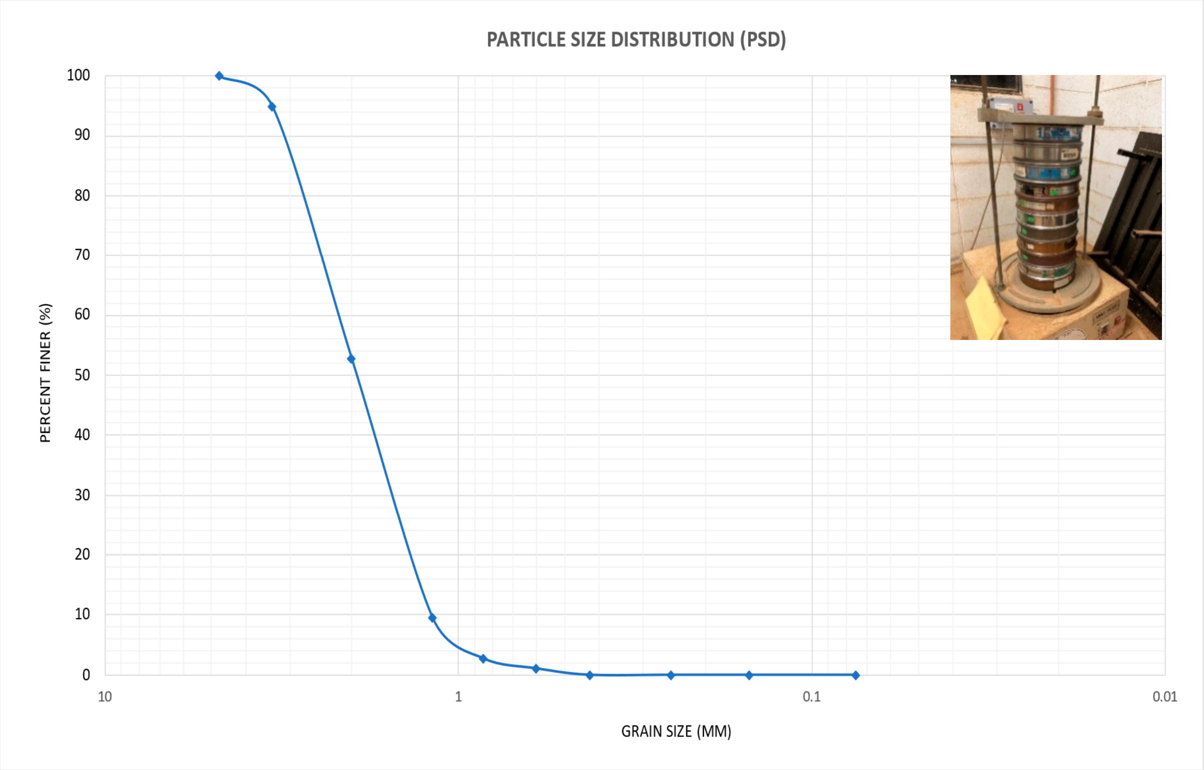

Figure S5 The grain size distribution curve of the sand was used in this study for the biocementation test.

Table S1. Physicochemical characterization of leachate sample.

Parameter	Unit	Result
Total suspended solids (TSS)	mg/L	340
Dissolved oxygen	mg/L	0.31
Turbidity	mg/L	378
Chemical oxygen demand (COD)	mg/L	78700
Biological oxygen demand (BOD_5)	mg/L	24000
Nitrate	mg/L	27
Nitrite	mg/L	1000
Ammoniacal nitrogen	mg/L	2000
Electric conductivity	mS/cm	27.84
pH		6.5
Total dissolved solids (TDS)	mg/L	1200
Lead	mg/L	0.15
Cadmium	mg/L	0.08
Phosphate (PO_4)	mg/L	30
Sulfate (SO_4)	mg/L	200
Chloride (Cl)	mg/L	800
Total Organic Carbon (TOC)	mg/L	4000
Absorbance at 305.5 nm	Abs	4.00
Absorbance at 594.0 nm	Abs	0.30
